# Supplementary material for: Multicolor fluorescence fluctuation spectroscopy in living cells via spectral detection
Source: eLife. 2021 Sep 8;10:e69687. doi: 10.7554/eLife.69687 (PMC8545396; doi:10.7554/eLife.69687)
Supplement: Supplementary file 1. — (a): Linker sequences of FP hetero-oligomer constructs. (b): Day-to-day variability of molecular brightness values obtained from three-species RSICS measurements. [file elife-69687-supp1.docx]

**Supplementary file 1a. Linker sequences of FP hetero-oligomer constructs.**

| **Plasmid** | **Linker sequence between FPs** |
| --- | --- |
| mp-mEGFP-mEYFP | LK |
| mp-mEYFP-mEGFP | PPAAAPPVLSLVP |
| mp-mCherry2-mEGFP | SGLRSRG |
| mp-mEYFP-mCherry2-mEGFP | 1^st^: PPAAAPPVLSLVP,  2^nd^: PPAAAPPVVP |
| mp-mEYFP-mCherry2-mEGFP-mApple | 1^st^: PPAAAPPVLSLVP,  2^nd^: PPAAAPPVVP, 3^rd^: PPAAAPPVDP |
| mp-mCherry2-mApple | PPAAAPPVVP |
| mCherry2-mEGFP | SGLRSRG |
| mEYFP-mApple | PPAAAPPVLSLVPSS |
| mEYFP-mCherry2-mEGFP | 1^st^: PPAAAPPVLSLVP,  2^nd^: PPAAAPPVVP |
| mEYFP-mCherry2-mEGFP-mApple | 1^st^: PPAAAPPVLSLVP,  2^nd^: PPAAAPPVVP, 3^rd^: PPAAAPPVDP |

**Supplementary file 1b. Day-to-day variability of molecular brightness values obtained from three-species RSICS measurements.** Normalized brightness values for 2x-mEGFP, 2x-mEYFP, and 2x-mCherry2 were obtained by normalization to the average brightness values detected for free monomers, on the same day.

| **Date** |  | **Normalized brightness** | | | **Absolute brightness [kHz]** | | |
| --- | --- | --- | --- | --- | --- | --- | --- |
|  | Sample | 2x-G | 2x-Y | 2x-Ch2 | 1x-G | 1x-Y | 1x-Ch2 |
| 16.06.20 | mean | 1.59 | 1.77 | 1.58 | 21.9 | 14.5 | 6.30 |
|  | median | 1.62 | 1.79 | 1.51 | 21.8 | 14.4 | 6.29 |
|  | SD | 0.23 | 0.22 | 0.20 | 3.1 | 1.7 | 0.71 |
|  | n | 9 | 9 | 9 | 14 | 14 | 14 |
|  |  |  |  |  |  |  |  |
| 14.10.20 | mean | 1.82 | 1.78 | 1.67 | 17.8 | 12.5 | 6.62 |
|  | median | 1.79 | 1.71 | 1.67 | 17.5 | 12.3 | 6.61 |
|  | SD | 0.44 | 0.38 | 0.34 | 3.8 | 2.8 | 1.21 |
|  | n | 14 | 14 | 14 | 14 | 14 | 14 |
|  |  |  |  |  |  |  |  |
| 10.07.20 | mean | 1.78 | 1.76 | 1.57 | 17.4 | 11.6 | 5.88 |
|  | median | 1.66 | 1.77 | 1.54 | 17.6 | 12.1 | 5.88 |
|  | SD | 0.37 | 0.36 | 0.29 | 2.0 | 1.5 | 0.58 |
|  | n | 16 | 16 | 16 | 14 | 14 | 14 |
